# Strategies for Reversing Exogenous Testosterone-Induced Infertility

**DOI:** 10.7759/cureus.91975

**Published:** 2025-09-10

**Authors:** Robert Liberto, Nachum Katlowitz, Daniel Sagalovich, Jonathan Davila

**Affiliations:** 1 Urology, Northwell Health, Staten Island, USA

**Keywords:** hpg axis, hypogonadism, infertility, spermatogenesis, testosterone

## Abstract

Exogenous testosterone use in men of peak fertility age is a particular concern, as it can lead to disruption of the hypothalamic-pituitary-gonadal (HPG) axis, resulting in infertility. Unfortunately, this population is often unaware of the adverse events, specifically infertility, that can arise from exogenous testosterone use. Discontinuation of exogenous testosterone may result in recovery in the HPG axis with time; however, not all patients will rebound or tolerate symptoms of low testosterone in the interim. Additionally, some of these patients may have an immediate interest in conception. To date, there is a paucity of literature dealing with this growing population. This article aims to review infertility and HPG axis disruption resulting from exogenous testosterone use, examine pharmacological management, guide clinicians on how to approach these patients, and provide recommendations on when to refer to urology/andrology or endocrinology.

## Introduction and background

The prevalence of exogenous testosterone use by men of peak fertility age is rapidly increasing [1-3]. This phenomenon is multifactorial, including the rise of men's health clinics, the ability to easily obtain testosterone products over the internet, social media influence, and direct-to-consumer promotion of “testosterone boosting” and “male enhancement” products [4-6]. Unfortunately, these products are often used outside of intended indications without appropriate clinical consultation or consideration regarding potential adverse events. Exogenous testosterone abuse is a particular concern for men of reproductive age, as misuse can lead to a disruption of the hypothalamic-pituitary-gonadal (HPG) axis, resulting in the reduction or cessation of spermatogenesis and directly impacting fertility [7].

Presently, there are no standardized guidelines on how to manage patients with a history of exogenous testosterone abuse, specifically concerning testosterone-induced infertility. Clinicians encounter additional challenges in identifying men with sequelae of prior exogenous testosterone use due to perceived stigmas, compelling men to seek treatment outside of official channels [4]. As more clinicians encounter younger men using exogenous testosterone, it is necessary to conceptualize its effect on spermatogenesis, understand the risk associated with infertility, and review methods to restore the HPG axis.

The hypothalamic-pituitary-gonadal axis

**Figure 1:**
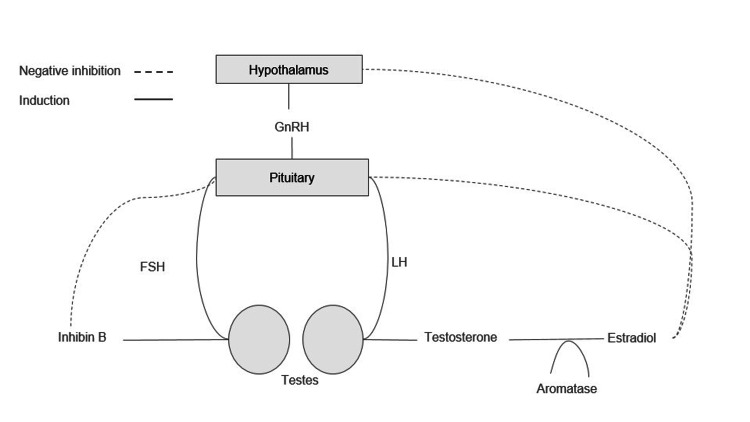
Hormonal regulation of the hypothalamic-pituitary gonadal axis Image Credit: Robert Liberto GnRH: Gonadotropin-releasing hormone; FSH: Follicle-stimulating hormone; LH: Leutenizing hormone

The HPG axis is a complex regulatory pathway responsible for spermatogenesis and testosterone production, as shown in Figure 1. The hypothalamus secretes gonadotropin-releasing hormone (GnRH) in a pulsatile fashion, which stimulates the release of both luteinizing hormone (LH) and follicular-stimulating hormone (FSH) from the anterior pituitary [3,8,9]. FSH acts on the Sertoli cells of the seminiferous tubules of the testicles to promote spermatogenesis [10]. During spermatogenesis, the Sertoli cells release inhibin B, a glycoprotein that provides negative feedback inhibition of FSH [11].

LH facilitates the production of testosterone within the Leydig cells of the testicle [10]. Testosterone is converted to estradiol via aromatization, which in turn acts as negative feedback inhibition to the production of LH [12,13]. Moreover, estradiol exerts inhibitory effects on spermatogenesis by downregulating genes related to sperm formation [13]. Furthermore, testosterone can independently act as a negative feedback inhibition to the release of GnRH [8]. Testosterone can be further converted to dihydrotestosterone (DHT) by 5-alpha reductase. DHT plays a critical role in prostatic growth and secretions, which aids in the formation of semen [8].

The process of spermatogenesis is dependent on the complex relationship between testosterone and FSH. To support the proper development and maturation of sperm cells, it is critical to sustain elevated levels of testosterone within the testicles, which can be significantly higher, by up to 50-100 times, than the levels found in the bloodstream [3,8,9]. Hence, both FSH and LH are crucial factors in spermatogenesis, and disruption in either gonadotropin can lead to impaired production and maturation.

The effects of testosterone on spermatogenesis

Exogenous testosterone can come in a variety of forms, including injectables, topicals, patches, intranasal, and oral medications [14]. While all forms of exogenous testosterone affect the HPG axis, some formulations may have a more substantial impact within a shorter timeframe such as injectables [3,5,6]. In contrast, topical and intranasal formulations may take longer to cause an effect [6,9]. However, regardless of the formulation, it is possible that men can experience a significant impact on spermatogenesis from exogenous testosterone use as early as 10 weeks after starting therapy, with 65% of men developing azoospermia at 4-6 months [1,3,6].

Exogenous testosterone causes depression of the HPG axis by direct suppression of GnRH, leading to the decrease and eventual cessation of LH and FSH release [3,5,8,9]. Additionally, excess aromatization of exogenous testosterone can lead to higher estradiol levels, which also act in an inhibitory fashion to LH [1,2]. This reduction in LH causes intratesticular testosterone levels to decrease up to 94% from normal to suboptimal levels, and without sufficient FSH to trigger the process, spermatogenesis becomes significantly impaired [1].

Recovery of the HPG axis and normal spermatogenesis after cessation of exogenous testosterone use is variable and linked to multiple factors, including length of use, dosage, and formulation [5,12,13]. Recovery from short-term use, less than one year, is typically possible without the aid of pharmacological agents within four to six months [1,13,15]. However, patients with a history of long-term or high-dose dosage that increases serum testosterone levels above the normal physiologic range can take as long as two years and often need pharmacological management to help restore the HPG axis [13,15]. Additionally, approximately 10-20% of men will have no recovery of the HPG axis or spermatogenesis after discontinuation of exogenous testosterone [1,13].

## Review

Pharmacologic agents

The pharmacologic management of infertility due to exogenous testosterone is controversial and not well-studied. There is scant literature dealing with this particular patient population, and treatment strategies are based on the management of general male infertility, specifically hypogonadotropic hypogonadism, which closely mirrors the state caused by exogenous testosterone use [16]. To date, there are no official recommendations from any professional societies regarding restoring the HPG axis after exogenous testosterone use other than the discontinuation of exogenous testosterone in men who wish to pursue conception [9]. Typical methods rely on discontinuing exogenous testosterone and employing methods to increase FSH and LH production from the pituitary to return serum and intratesticular testosterone to normal physiologic levels and improve sperm parameters [13]. All pharmacological agents used, shown in Table 1, are in an off-label fashion and not approved by the U.S. Food and Drug Administration (FDA) for this patient population.

**Table 1:** Pharmacologic agents SERMs: Selective estrogen receptor modulators; AI: Aromatase inhibitors; hCG: Human chorionic gonadotropin; FSH: Folicular stimulating hormone; LH: Leutinizing hormone; BMD: Bone mineral density; HCT: Hematocrit

	Medication	Dosage	Effect	Adverse Events
SERMs	Clomiphene Citrate	25 mg - 50 mg PO every other day or daily	Increase in total testosterone, FSH, and LH. Improved spermatogenesis	Gynocomastia, weight gain, hair loss GI upset, visual disturbances
	Enclomiophene Citrate	12.5-25 mg PO daily		
AIs	Anastrazole	1 mg PO daily	Increased in total testosterone, FSH, and LH. Improved spermatogenesis. Decreased estradiol	Elevated liver enzymes, headaches, decreased BMD
	Letrozole	2.5 mg PO daily		
Gonadotropins	hCG	1500-2000 IU, IM or SubQ injection, two to three times weekly	Increase in total testosterone	Gynocomastia, elevated HCT

Selective Estrogen Receptor Modulators

Selective estrogen receptor modulators (SERMs) competitively inhibit estrogen at the estrogen receptors in the hypothalamus and pituitary [13,17,18]. As a result, the negative feedback effect of estrogen is blunted, leading to a greater release of GnRH, LH, and FSH [16,18].

Clomiphene citrate (CC) is the most widely used SERM for male infertility [8]. It is administered orally at 25-50 mg every other day and can be titrated for daily use based on laboratory results [17]. A systematic review by Huijben et al. found significantly improved FSH, LH, and total testosterone with improvement in sperm concentration and motility in men with infertility for various reasons, including idiopathic male infertility and hypogonadotropic hypogonadism, treated with CC in a meta-analysis of over 200 patients [18]. 

In a review by Khourdaji et al., they reported that in 53 males with idiopathic infertility placed on CC, 50% had a dramatic improvement in FSH, LH, and testosterone levels as early as 3 months after initiating treatment, with the remainder responding within 6-15 months of treatment [13]. Furthermore, fifty-six hypogonadotropic men with documented low FSH levels administered CC 50 mg daily had marked improvement in sperm parameters, including volume and motility, at nine months of treatment [13]. 

Enclomiphene citrate (EC), an isomer of CC, has been used for both male infertility and testosterone replacement [7,8]. While CC has antagonistic effects on estrogen receptors, it also exhibits some agonist effects and may paradoxically negatively affect sperm parameters, especially with long-term use [7,8]. In contrast, EC has no agonistic effects and has been theorized to be superior to CC [7]. Thomas et al. performed a retrospective analysis of CC versus EC on seventy-eight infertile males and found similar elevations in serum testosterone but improved FSH, LH, and total motile sperm counts as compared to CC [7].

SERMs are typically well-tolerated, with the most common adverse events being gynecomastia, hair loss, weight gain, and gastrointestinal upset [13,18]. Rare visual disturbances are possible; however, they are reversible with cessation of therapy [13].

Aromatase Inhibitors

Aromatase inhibitors (AIs) inhibit the aromatase enzyme, which is responsible for converting testosterone to estrogen [16]. Aromatase is primarily found in adipose tissue, liver, brain, and testes [8]. Inhibiting aromatase has a dual effect in that blocking the conversion of testosterone to estrogen increases the amount of available serum testosterone, which is often depleted in men with a disrupted HPG axis [13,17]. Additionally, the reduction of estrogen leads to an increase in GnRH, LH, and FSH, as the lack of feedback inhibition in the absence of estrogen triggers this hormonal cascade [8].

Two of the most commonly used AIs are anastrozole and letrozole [17]. Studies demonstrate that anastrozole, dosed orally at 1 mg per day, improved sperm concentration and motility in subfertile men [13]. This effect is more pronounced in obese men, likely due to increased aromatase conversion [13,17]. Similarly, letrozole, orally, at 2.5 mg per day, was found to improve sperm concentration and motility in 27 azoospermic and oligospermic men after 6 months of treatment [13]. Moreover, letrozole has been found to increase LH, FSH, and total testosterone levels in idiopathic azoospermic men one month after treatment [13].

Helo et al. performed a randomized prospective study comparing the effects of CC and anastrozole on 26 hypogonadal infertile men [19]. Although both groups responded with increased serum testosterone levels, there was a more remarkable rise in serum testosterone from baseline in patients receiving CC at 12 weeks than in patients in the anastrozole group.

Adverse events associated with AIs are generally mild and include headaches and elevated liver enzymes, which resolve with discontinuation of therapy [13]. It is important to note that estrogen is essential for bone health; therefore, there is a risk of decreased bone mineral density when taking AIs [16]. Additionally, there is some concern about an increased risk of venous thromboembolism based on studies involving women with breast cancer who were receiving treatment with AIs; however, the risk in men is unclear [16].

Gonadotropins

Human chorionic gonadotropic hormone (hCG) is a commonly prescribed treatment for men with hypogonadotropic hypogonadism [13]. This hormone is composed of two subunits, alpha and beta [1]. The alpha subunit is analogous to LH, while the beta subunit has been found to enhance receptor activity in the Leydig cells [1]. The beta subunit is responsible for hCG's half-life of 36 hours, compared to LH's half-life of 30 minutes [1]. The typical dosage of hCG is between 1,500 and 3,000 IU intramuscular injection two to three times per week [1,17].

A review of the literature by Lee and Ramasamy focused on hCG in the recovery of spermatogenesis following exogenous testosterone use [1]. Their findings revealed that hCG, dosed at 3,000 IU every other day, in combination with either CC, anastrozole, or an FSH analog, resulted in the restoration of spermatogenesis within 4 months of therapy [1]. Moreover, in men with hypogonadotropic hypogonadism, hCG dosed between 1,500-2,000 IU two to three times weekly normalized testosterone levels within 4-6 months [1].

While hCG has demonstrated efficacy at increasing and normalizing testosterone levels, it is sometimes not sufficient on its own in restoring spermatogenesis. This is because hCG functions primarily as an LH analog, while spermatogenesis requires both LH and FSH [16]. In hypogonadotropic hypogonadal men, recombinant FSH (rFSH) has been used in conjunction with hCG to stimulate spermatogenesis when hCG alone was unsuccessful [8]. According to a review by Desai et al., a combination of hCG and rFSH resulted in the restoration of spermatogenesis in 90% of patients in two trials of eighty-seven and seventy-five hypogonadotropic hypogonadal men [8]. The adverse event profile of hCG is minimal. The most common adverse events are gynecomastia and, rarely, elevated hematocrit [16].

Clinical application

Exogenous testosterone use poses a challenge for clinicians since these patients may acquire testosterone from either a non-clinical source or men's health clinics and may not disclose their use during their visits [20]. It is the author's opinion that clinicians should consider screening men of peak fertility age for a history of or current exogenous testosterone use if, in their visit, they discuss difficulty with conception, new-onset behavior changes, or preoccupation with exercise or sexual performance. Moreover, questions of testosterone use should be brought up if routine physical examination demonstrates atrophic testicles, gynecomastia, or truncal acne [20]. If routine lab tests reveal elevated hemoglobin or hematocrit levels, clinicians should inquire about testosterone usage since polycythemia is a common adverse event [2].

It is important to foster a safe and accepting dialogue with these patients and focus on understanding the patient's reasons for using testosterone [20]. It is recommended to review the adverse events, specifically infertility, as these patients may be generally unaware of the risk. In cases where the patient's concern is around muscle mass or physical activity, the clinician should discuss natural ways of increasing testosterone, such as maintaining a healthy diet and exercise routine, as well as a suitable sleep schedule of at least 8 hours per day [21]. The patient should be consulted to discontinue exogenous testosterone therapy, especially if there are any concerns regarding infertility [1,5]. 

Initially, the clinician should order laboratory work consisting of total testosterone, FSH, LH, estradiol, complete blood count (CBC), prolactin, and a hepatic function panel [1]. If there is an immediate concern for fertility, then inhibin B and/or a complete semen analysis can be ordered, as FSH levels, together with inhibin B levels, are a strong predictor of spermatogenesis [11].

Discontinuing the use of exogenous testosterone may result in a decline in energy, libido, and erectile function [22]. It is important to counsel patients that these effects are temporary and will resolve once the HPG axis resets. If the patient has been using testosterone for less than one year, the HPG axis can return to normal without pharmacologic intervention [15]. However, patients who are unable to tolerate these symptoms can consider starting CC [23]. Total testosterone, FSH, LH, estradiol, CBC, and hepatic function panel should be requested at four to six weeks of therapy, with the goal of achieving normal total testosterone, FSH, and LH levels. In cases where there is a poor response to treatment or continuation of these symptoms despite a return to normal physiologic testosterone levels, consider urology/andrology or endocrinology consultation.

For patients with long-term exogenous testosterone use, where fertility is not an immediate concern, it is expert opinion to obtain a baseline semen analysis and start CC 50 mg every other day along with hCG 2,000 IU every other day [1]. Total testosterone, LH, FSH, estradiol, hepatic function panel, and CBC at 4-6 weeks. If there is a poor response to treatment, consider referral. If there are elevated estradiol levels, consider adding 1 mg of anastrozole twice weekly and recheck labs in 6 weeks [1]. Repeat semen analysis should be obtained at three months [1]. Refer for expert consultation with andrology/urology if there is a suboptimal response to treatment, oligospermia or azoospermia at three months, or continued symptoms despite the return of normal hormone levels.

Regardless of exogenous testosterone length of use, if infertility is a primary concern or the patient is trying to conceive within six months to one year, and initial semen analysis demonstrates oligospermia or azoospermia, expert consultation should be obtained. These patients may need initial therapy with multiple medications, including rFSH, a tight titration schedule, and close observation.

## Conclusions

As more clinicians encounter men of peak fertility age using exogenous testosterone, it is important to understand ways to restore the hypothalamic-pituitary-gonadal (HPG) axis and improve the chances of fertility. These patients should be counseled to discontinue exogenous testosterone use with an open, non-judgmental approach and be given proper direction on methods to restore the HPG axis. There is a considerable lack of literature dealing with this particular patient population, and more studies and guidance from professional societies are needed. Additionally, professional societies should consider statements against obtaining exogenous testosterone and other male enhancement products via online sources without proper medical evaluations. The use of SERMs, AIs, and gonadotropins has been shown to assist in the rehabilitation of the HPG axis in hypogonadal men. Although there is limited research on this patient population, these treatments may also help restore fertility in cases of hypogonadism induced by exogenous testosterone.
